# What factors play a role in preventing self-immolation? Results from a case-control study in Iran

**DOI:** 10.5249/jivr.v7i2.550

**Published:** 2015-07

**Authors:** Hosein Karim, David C. Schwebel, Shahrzad Bazargan-Hejazi, Reza Mohammadi, Mansour Choubsaz, Zahra Heidari Zadie, Alireza Ahmadi

**Affiliations:** ^a^Department of Cardiology, Imam Ali Hospital, Kermanshah University of Medical Sciences, Kermanshah, Iran.; ^*b*^Department of Psychology, University of Alabama at Birmingham, USA.; ^*c*^Department of Psychiatry, College of Medicine, Charles Drew University of Medicine and Science, Los Angeles, CA. USA.; ^*d*^Semel Institute for Neuroscience and Human Behavior, David Geffen School of Medicine, UCLA , CA. USA.; ^*e*^Department of Public Health Sciences, Division of Social Medicine, Karolinska Institute, Stockholm, Sweden.; ^*f*^Department of Anesthesiology, Critical Care and Pain Management, Imam Reza Hospital, Kermanshah University of Medical Sciences, Kermanshah, Iran.

**Keywords:** Case control, Self-immolation, Impulsivity, Counseling services, Burn

## Abstract

**Background:**

To investigate factors related to prevention of self-immolation in west of Iran.

**Methods:**

In a case-control study, 30 consecutive cases of deliberate self-inflicted burns admitted to the regional burn center (Imam Khomeini hospital in Kermanshah province, Iran) were compared with controls selected from the community and matched by sex, age, district-county of residence, and rural vs urban living environment. The following characteristics relevant to preventing self-immolation were collected from all cases and controls: main domestic fuel used in the household, awareness about complications of burn injuries, and use of counseling services.

**Results:**

Descriptive analyses revealed that kerosene was the main domestic fuel in the household for 83% of cases. Not surprisingly, the main means of self-immolation in 93% of the patients was kerosene, with other fuels such as petrol and domestic gas used in remaining cases. The majority of cases and controls were aware of the potential complications of burn injuries. Use of counseling services was more common in controls.

**Conclusions:**

All three aspects of preventing self-immolation – having kerosene and other fuels in the home, being aware of the complications of burn injuries, and using counseling services were present in both the cases and controls. This suggests a large portion of residents in rural Iran are potential self-immolation victims. Increasing preventive strategies may reduce risk of suicide by self-immolation.

## Introduction

It is estimated that approximately 4000 people commit suicide in Iran on a yearly basis. Of those, almost one-quarter are victims of self-immolation (self-burning),^1^ a method of committing suicide that is rare in high-income countries^2-7^ but common in low- and middle-income countries.^8^ Iran is among the countries with the highest rates of self-immolation. In fact, some regions of Iran have the highest documented rates of self-immolation in the world (22.4 per 100,000 person-years). ^9-12^

As a result, prevention of self-immolation has become a public health priority in Iranian society. One public health approach for suicide prevention suggests the following five phases: ^13^

(1) Define the problem: conduct surveillance; 

(2) Identify the cause: complete risk and protective factor research; 

(3) Develop and test interventions;

(4) Implement interventions; and 

(5) Evaluate effectiveness of interventions.

Our previous work, along with that of others,^8,10-12^ has accomplished steps 1^1,2,14^ and 2.^15-19^ Our findings indicate individuals at greatest risk are younger women with a history of financial hardship, break-ups in intimate relationship and previous suicide attempts,^16-19^ as well as depression and adjustment disorders. Among men, opiate dependency is associated with risk.^15^ Consistent with scientific calls to study prevention as well as risk for human dysfunction,^20^ the current study extends beyond an investigation of risk factors to understand factors that are associated with prevention of self-immolation.

## Methods

**Participants**

Thirty Patients admitted to the regional Burn center (Imam Khomeini hospital in Kermanshah province, Iran) because of deliberate self-burning were enrolled consecutively. We included in this study only those patients who clearly and unequivocally attempted self-immolation with suicidal intent. This evidence came from the patient’s confession to deliberate self-burning and/or reports from reliable witnesses. Patients whose suicide seemed suspicious (i.e., those who denied suicidal intent and for whom there were no corroborating witnesses or data) were excluded. 

The control group was recruited from the community. Studies consistently report elevated risk of self-immolation among late-adolescent and young adult women in Iran.^1,8-12,14,19^ As a result, we matched these factors between case and control group. We also matched by district-county of residence and living in rural vs. urban regions given the possibility for people living in different settings to be exposed to different prevention-oriented risk factors (e.g., access to fuels).

**Protocol**

A clinical psychologist interviewed all self-immolation patients within the first 24 hours of hospital admission. Information for the study was collected directly from patients via semi-structured interview self-report in all but 3 cases. For those 3 individuals, all of whom had severe burning (>90% Total Body Surface Area (TBSA)), information was collected from spouses or parents.

**Outcome variables on prevention**

Several strategies have been suggested for suicide and self-immolation prevention. We focused on three of the most prominent: (a) restricting access to means of suicide, such as fuel for self-immolation, (b) educating victims about the consequences of a suicide attempt (both successful and failed), and (c) providing mental health counseling and promotion. 

We conceptualized mental health counseling to include strategies like victim stories-based intervention,^14^ building resilience through peer-support and awareness of suicidality, and behavioral interventions. In all cases, counseling is likely to address issues of imitating suicide attempts by others; improving mental health to overcome depression, adjustment problems and other symptoms of mental illness; and developing appropriate peer and family support networks.^1,2,13,14,21-24^

**Measures**

Informed by previous work, we examined the preventive role of the following factors in the sample as well as among both cases and controls in the sample:

1. Whether means for self-immolation, such as fuels, were available (yes vs. no)

2. Knowledge of burn injury-related complications (yes vs. no)

3. Recent professional counseling services (yes vs. no)

4. Among victims, the primary means by which self-immolation was performed 

The entire protocol was approved by the Kermanshah University of Medical Sciences (KUMS) Research Ethics Committee, and all participants (or their legal proxies) provided informed consent to participate in the research.

**Analytical method**

The analysis was undertaken in two steps. First, we examined descriptive data for all outcomes. Second, we computed a series of chi-square and odds ratio analyses to estimate the difference and strength of the difference between outcome prevention-relevant variables and case vs. control status. A p-value of 0.05 was used throughout the study.

## Results

Table 1 lists characteristics of the sample. We also found that the primary means of self-immolation was kerosene, which was used by 28 (93%) patients in their self-immolation act. The remaining 2 (7%) patients used other fuels such as petrol or domestic gas. Over half of the sample (57%) reported imitating this method of suicide from someone else (Data not shown).

**Table 1:** Demographic data of case (n=30) and control (n=30) groups.

Variable	Groups		
	Cases	Control	Total
**Gender; N (%)**			
Male	4 (13)	4 (13)	8 (13)
Female	26(87)	26(87)	52(87)
**Marital state; N (%)**			
Single	12 (40)	10(33)	22(37)
Married	17(57)	19(64)	36(60)
Divorced	1(3)	1(3)	2(3)
Mean of age;(year)	27.5	28.5	28.0
Mean of TBSA*; (%)	60.2	-	-

* Total Body Surface Area

As shown in Table 2, bivariate comparisons revealed that all participants in both the case and control groups had easy access to the means of self-immolation, especially to kerosene fuel. Kerosene was the primary domestic fuel used by both the cases (n = 25, or 83%) and controls (n = 22, or 73%), and the difference between the two groups was not statistically different (x2 = 0.88, p=0.35, Odds Ratio (OR) = 1.82, 95% Confidence Interval (CI) =0.52-6.38) (Table 1).

**Table 2:** Difference between self-immolation and variables (cases n =30; controls n=30).

	x2	p-value	Odds Ratio	95% CI
**Access to kerosene as the primary domestic fuel used in the family**	0.88	0.35	1.82	0.52-6.38
**Knowledge about potential complications of burn injuries**	2.96	0.09	5.80	0.64-53.01
**Recent use of professional counseling services**	2.78	0.10	3.27	0.77-13.83

Most participants were knowledgeable about the potential medical complications of burn-related injuries (n = 29, or 97% in the case group and n = 25, or 83% in the control group). This difference was not significant (x2 = 2.96, p=0.09, OR = 5.80, 95% CI =0.64-53.01) (Table 2).

A smaller percentage of cases (n=3, 10%) reported using professional counseling services recently than in the control group (n= 8, 27%). Although in the opposite direction of what we expected, this difference was not statistically significant (x2 = 2.78, p=0.10, OR = 3.27, 95% CI =0.77-13.83) (Table 2).

Since it is relevant to prevention, we also asked victims whether they had pre-planned their self-immolation. Unplanned or impulsive self-immolation was reported in 24 (80%) of the patients. Further investigation revealed that, just one of these 24 patients was male (4%) and 23 or 96% were female. The difference between the genders was statistically significant (x2 = 3.90, p<0.05, OR = 7.67, 95%).

## Discussion

Findings from this study confirm that kerosene is the primary means of self-immolation used by self-immolation victims in Iran. Kerosene is highly flammable and readily available, inexpensive, and accessible in many low to middle income countries.^25,26,27^ As countries move towards modernization, the availability and usage of kerosene as a means of suicide is expected to drop. In the meantime, communities where kerosene is a primary household fuel should become more mindful of proper usage and handling. At the community level, the marketing and usage of such fuel should be regulated by policy.

The majority of the patients in this study reported choosing self-immolation as a method of suicide in an impulsive manner, often because they were imitating someone else in their communities. Such “copycat” behavior is common in suicidality^28^ and occurs despite the fact that participants recognize the consequences of suicide attempts and acts; for example, in this study both cases and controls were quite aware of the potential complications of burn-related injuries. Interventions where victims of self-immolation have shared their personal stories with their communities have shown promising results in modifying community’s perception regarding the imitational self-immolation. ^14^ These types of interventions also have potential to enhance individuals’ coping skills.^14^

The literature on suicide prevention suggests counseling and therapy are fairly effective in reducing suicide risk.^29^ Our findings, however, revealed that participants in the control group were more frequent users of these services than the cases. Of those who did use counseling, it was usually peer counseling rather than using professional experts. Unfortunately, our data lacks information on frequency, quality, duration or time of counseling services. Further research, including interventional studies, should evaluate the extent to which the burden of self-immolation might be reduced by enhancing and institutionalizing the culture of using professional counseling services in local communities. Such efforts may require raising community awareness regarding the negative consequences of unchecked daily strains and life stresses. Religious leaders could play an important role in motivating and mobilizing the local community to rise against this risk by participating in prevention programs and accepting counseling as an effective and culturally-accepted way to cope with symptoms of mental illness and reduce impulsive acts of self-harm.

Beyond community and religious leaders, the role of mass media (radio, television) and curriculum development at the high school and college levels to help change cultural stigma regarding mental illness, disorder, and treatment needs further investigation. At the professional level, the role of training in screening and brief intervention and referral for treatment among primary health care providers should be considered. 

This study revealed that almost all female patients who attempt self-immolation do so in an unplanned, impulsive manner. Men are less likely to self-immolate, but when they do it appears to be carefully planned over time. Given the risks for women, creation of self-immolation prevention centers that address specific needs of women may be valuable. Such centers might offer counseling, outreach, and telephone hotlines, and might focus especially on cultural aspects of women’s role in society, mental health symptoms, and marital discord and relations. In addition, self-immolation preventive programs may benefit from research that investigates gender roles as it relates to women’s resiliency, especially from impulsive or aggressive acts at times of stress. Findings from such foundational research could educate programs that aim to enhance the psychosocial coping capacity of women at risk for self -immolation. They also could target men who have organized plans for self-immolation. 

**Limitations**

As a pilot study, we recognize the limitations of this study. It was implemented in a geographically limited area in Iran and findings may not generalize. The sample was somewhat large for a clinical sample, but still small to accomplish sufficient statistical power to detect differences in many of the analyses we conducted.

Despite these limitations, this is the first case-control self-immolation study conducted in a region of Iran with very high self-immolation rates. Our findings offer information that may benefit development and implementation of self-immolation prevention strategies in Iran and beyond.

## Conclusion

Our results indicate that kerosene is highly available and is the primary means of self-immolation in this region in Iran. Moreover, the majority of the self-immolation victims were aware of the consequences of burn injuries but still committed self-immolations in an impulsive, unplanned manner. Our findings suggest that unplanned self-immolation is more common in women than men. We also found that very few people in either the case or control group used counseling services, but counseling services were accesses more often in the controls than in the patients. Several interventional strategies at the individual, system and policy levels were suggested for further investigation.^30^
